# Predictors of HIV status disclosure among people living with HIV (PLHIV) in Ghana: the disclosure conundrum and its policy implications in resource limited settings

**DOI:** 10.1186/s12981-023-00569-1

**Published:** 2023-11-27

**Authors:** Robert Kaba Alhassan, Jerry John Nutor, Akua Gyamerah, Emily Boakye-Yiadom, Emmanuel Kasu, Evelyn Acquah, Emmanuel Doe

**Affiliations:** 1 Institute of Health Research, University of Health and Allied Sciences, PMB 31, Ho, Ghana; 2 University of California San Francisco School of Nursing, San Francisco, USA; 3 School of Medicine, University of Health and Allied Sciences, Ho, Ghana; 4 Ho Teaching Hospital, Ministry of Health (MoH), Ho, Ghana

**Keywords:** HIV, AIDS, Status self-disclosure, Antiretroviral therapy, People living with HIV (PLHIV), Policy, Low-and-middle-income countries (LMICs), Africa, Sub-Saharan Africa, Ghana

## Abstract

**Background:**

Globally, over 40 million lives have been claimed by HIV/AIDS. In Ghana, more than 350,000 people are living with HIV. Non-disclosure of HIV status is a major barrier to HIV/AIDS eradication; yet, little is known of the determinants of HIV status disclosure in resource limited settings in Africa like Ghana.

**Objective:**

Determine the predictors of HIV status disclosure among people living with HIV (PLHIV) and stimulate policy discourse on support systems for self-disclosure in Africa.

**Methods:**

This is a descriptive cross-sectional study among PLHIV (n = 181) in sub-Saharan Africa, specifically the Volta region of Ghana. Bivariate probit regression was run to determine factors associated with HIV status disclosure among PLHIV.

**Results:**

HIV status self-disclosure was reported by 50% of the respondents; nearly 65% disclosed their status to non-family members and non-partners. Significant correlates of HIV status disclosure either to partners or non-partners were marital status, monthly income, type of occupation, and being divorced due to HIV status (p < 0.05).

**Conclusions:**

HIV status disclosure remains low in Ghana like many African countries. There is the need for a renewed policy debate on tailored guidelines for HIV status self-disclosure and targeted support systems for PLHIV to ameliorate their predicaments and promote eradication of the epidemic in Africa.

## Background

According to the World Health Organization (WHO) [23], over 40 million lives have so far been claimed by HIV/AIDS globally out of over 75 million infected persons. At the end of 2022 an estimated 39 million people were living with HIV and two thirds of those infected lived in the WHO-African region [23]. Per the WHO 2025 target, 95% of all people living with HIV (PLHIV) should have a diagnosis, 95% of those diagnosed should be taking lifesaving antiretroviral treatment (ART) and 95% of PLHIV on treatment achieving a suppressed viral load. However, the current global statistics stand at 86%, 76% and 71%, respectively.

Over the years, substantial number of studies have investigated outcomes of HIV status disclosure [2, 3, 6, 17]. Unfortunately, little is known on the determinants of HIV status self-disclosure among PLHIV. This study investigated the predictors of HIV status self-disclosure among PLHIV to inform national policy discourse on support systems for self-disclosure as a conduit for HIV/AIDS control.

## Methods

### Study design

The study is a descriptive cross-sectional design that employed survey approach to ascertain status self-disclosure and the predictors among PLHIV.

### Study setting and sampling procedure

The study was conducted in the Anti-retroviral Therapy (ART) clinic of a tertiary referral hospital in Ghana. The study population was PLHIV aged 18 years and above who have ever attended the ART clinic at least once for the last 6 months. The sampling strategy was a census of adult PLHIV enrolled in ART and regularly attended the ART clinic.

### Sample size determination

Statistically, 60 patients per group (i.e. low and higher ART clinic attendant groups) was assumed to give the researchers the requisite statistical power, assuming a large effect size difference of 1.0 standard deviation units between the high and low ART clinic attendants groups. Subsequently, a conservative α = 0.01 adjusted from 0.05 was used to account for between 5 and 10 potential comparisons using mean differences (t-tests) or categorical differences (Chi-square) in individual and community factors associated with ART service utilization. In a linear regression model to account for the variance in ART clinic attendance rates, we simulated a standard normal distribution and 100–120 patients (about 10 cases per variable) and assumed to provide adequate power to achieve statistical significance (α = 0.05) [20]. Following the power calculation of the study, an over-sampling 61 respondents (representing 50%) was done to account for possible non-response or attrition due to the sensitive nature of the study. Thus, a final census of 181 eligible participants was done (n = 181) [16].

### Instruments of data collection and data sources

A structured questionnaire was developed and validated through piloting for the survey. An in-person survey was conducted among 181 PLHIV. For optimal privacy, one-on-one interviews were done in a private room for the participants. Each interview lasted approximately 45 mins. Data collection was between 14th and 30th June, 2021.

### Validity and reliability of test instruments

Computation of internal consistency reliability of the Likert’s scale items was done with a Cronbach’s alpha test and the mean scale reliability was  > 0.70.

### Data analysis

The data collected was analysed with the STATA statistical analysis software (version 16.0). All data sets were coded to anonymize the identity of respondents. Descriptive statistics were estimated in frequencies and percentages for categorical variables, and means/standard deviations for continuous variables. Cross-tabulation comparison of background information of respondents was done using Fisher’s Exact test. Bivariate probit regression tests were run to test the predictors of HIV status self-disclosure. Regression model outputs were reported in log likelihood ratios. Multicollinearity diagnostics were conducted on all explanatory variables and those with Variance Inflation Factors (VIFs) above 10.0 were excluded from the regression models.

### Outcome variables of interest

Main outcome variables were HIV status self-disclosure to a partner (yes = 1, no = 0), and self-disclosure to others (yes = 1, no = 0).

### Explanatory variables and co-variates

The socio-demographic explanatory variables were: marital status, education, sex, residence and religion. Other explanatory variables were monthly income, occupation, employment, divorce due to HIV, place of ART attendance and knowledge of ART side effects.

## Results

### Socio-demographic and economic characteristics of respondents

Total of 181 participants were recruited for the study and successfully interviewed. Females dominated, representing 80% of the respondents while the average age was 47 ± 12.6. Half of the respondents were urban residents while over 90% were Christians of varying denominations; more than 70% of the respondents either did not have formal education or had at most primary or secondary education. Out of the 44% of respondents who were employed, 40% were in private business employment with an average monthly income below GHC 500 (approximately USD 44.00); 69% of them perceived themselves as poor and not having enough in terms of their economic status (see Table 1).

**Table 1:** Demographic and socio-economic characteristics of respondents

Characteristics	Statistics	
Socio-demographic variables		
Age (n = 170)	Mean = 46.61, SD = 12.57; min = 18, max = 85	
Gender (n = 126)	Freq.	Percentage
Male	25	19.84
Female	101	80.16
Place of residence (n = 170)		
Rural	36	21.18
Urban	86	50.59
Peri-urban	48	28.24
District (n = 181)		
Adaklu	4	2.21
Adaklu Waya	2	1.10
Agotime-Ziope	11	6.08
Ho	151	83.43
Ho west	10	5.52
Hohoe	1	0.55
Ketu-north	1	0.55
North Dayi	1	0.55
Religious affiliation (n = 170)		
Protestant/Pentecostal	97	57.06
Catholic	21	12.35
Traditional	1	0.59
Other Christians	51	30.00
Education (n = 166)		
None	18	10.65
Primary	82	48.52
Secondary	48	28.40
Diploma/Degree	17	10.06
Marital status (n = 169)		
Widowed	37	21.89
Divorced	27	15.98
In relationship/living with a partner	71	42.01
In relationship/not living with a partner	22	13.02
Unmarried	12	7.10
Polygamous marriage (n = 165)		
Yes	6	3.64
No	159	96.36
Has children (n = 170)		
Yes	154	90.59
No	16	9.41
Number of children (n = 158)	Mean = 2.85, SD = 1.67; min = 1, max = 12	
HIV exposure proxies		
Any of the children has HIV (n = 151)		
Yes	20	13.25
No	125	82.78
Don’t know	6	3.97
Partner has HIV (n = 162)		
Yes	43	26.54
No	68	41.98
Never tested/Don’t know	51	31.48
HIV partner on ART (n = 42)		
Yes	34	80.95
No	8	19.05
Economic status proxies		
Occupation (n = 168)		
Student	5	2.98
Private business owner	67	39.88
Government/white collar job	10	5.95
Housewife	3	1.79
Farmer	27	16.07
Other	56	33.333
Employment status (n = 169)		
Employed	74	43.79
Unemployed	95	56.21
Monthly income (n = 155)		
Below 500 GHC	94	60.65
501–1,000 GHC	38	24.52
1,001–2,000 GHC	19	12.26
2,001–3,000 GHC	4	2.58
Perceived financial sufficiency (n = 170)		
Poor/not enough	117	68.82
Enough	53	31.18
Assets ownership (n = 170)		
Owns radio	111	65.29
Owns Television	101	59.76
Access to electricity	160	94.12
Economic status proxies (n = 170)		
Owns a car	16	9.41
Owns refrigerator	79	46.47
Access to potable water	143	84.12
Owns WC toilet	140	82.35
Owns solar electricity	3	1.76

Data source: Field Data (2021); n (valid responses)

*ART* Anti-retroviral Therapy, *HIV* Human Immune Virus, *SD* Standard Deviation

A little over 42% of respondents were in a  relationship and living with the partner. Over 90% of the respondents had at least a child with the average number of children per respondent being 3 ± 1.67. Approximately 83% of the respondents indicated none of their children was HIV positive; 42% of the respondents indicated their partner did not test positive for HIV and those whose partners were HIV positive, nearly 91% of them were on ART (see Table 1).

### HIV status disclosure among PLHIV

**Fig. 1:**
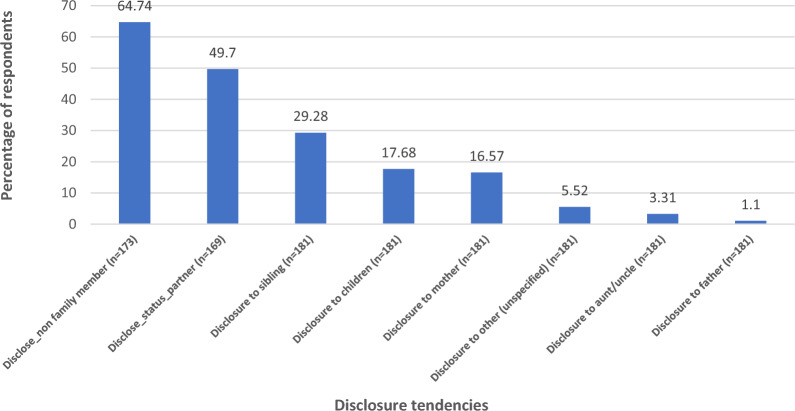
Key outcome variables of interest (HIV status self-disclosure)

Approximately 50% indicated they have not disclosed their HIV status to their partner while 50% of them said they did. Among respondents who did self-disclosure of their status, nearly 65% of them disclosed to non-family members followed by a partner (50%) and a sibling (29%). The least category of persons disclosures were made to are father (1.1%); aunt/uncle (3.3%); unspecific persons (5.5%); mother (17%), and children (18%) (see Fig. 1).

### Predictors of HIV status disclosure

Significant association was found between HIV status disclosure to a partner and socio-demographic factors such as sex (p = 0.046) and marital status (p = 0.000). Other significant correlates are: being in private business (p = 0.022) and divorced due to HIV (p = 0.000). HIV status self-disclosure to other persons other than the partner was significantly correlated with monthly income (p = 0.022), being employed (p = 0.027), being divorced due to HIV (p = 0.017) and being knowledgeable of ART drugs side effects (p = 0.044) (see Table 2).

**Table 2:** Relationship between HIV status self-disclosure and background characteristics of respondents

	Disclosure to partner (N = 84)				Disclosure to others (N = 112)			
Independent variables								
Socio-demographic variables	**Obs**	**No**	**Yes**	**p-value**	**Obs**	**No**	**Yes**	**p-value**
Age (Mean ± SD)	169	48 ± 13	45 ± 11	0.2713	173	44 ± 11	47 ± 13	0.0838*
	**Obs**	**f (%)**	**f (%)**	**p-value**	**Obs**	**f (%)**	**f (%)**	**p-value**
Females	98	58 (59)	40 (41)	0.046**	101	36 (36)	65 (58)	0.427
In a relationship with partner	73	17 (23)	56 (77)	0.000***	73	31 (42)	42 (58)	0.952
Pentecostal denomination	96	53 (55)	43 (45)	0.095*	99	34 (34)	65 (66)	0.447
Primary education	85	43 (51)	42 (49)	0.500	85	33 (39)	52 (61)	0.226
Urban resident	88	44 (50)	44 (50)	0.529	88	32 (36)	56 (64)	0.441
Economic variables								
Monthly income < GHC 500	93	51 (55)	42 (45)	0.017	96	27 (28)	69 (72)	0.022**
Poor financial situation	114	61 (54)	53 (46)	0.150	118	42 (36)	76 (64)	0.517
In private business	69	26 (38)	41 (59)	0.022**	69	27 (39)	42 (61)	0.227
Employed	92	51 (55)	41 (45)	0.081*	96	27 (28)	69 (72)	0.027**
HIV history variables								
Divorced due to HIV	73	54 (74)	19 (26)	0.000***	74	18 (24)	56 (76)	0.017**
Partner not HIV positive	66	35 (53)	31 (47)	0.271	68	25 (37)	43 (63)	0.421
Partner not on ART	36	2 (6)	34 (94)	0.145	36	18 (50)	18 (50)	0.404
Child not HIV positive	124	65 (52)	59 (48)	0.140	127	42 (33)	85 (67)	0.523
Takes ART at ART centre	128	63 (49)	65 (51)	0.376	131	42 (32)	89 (68)	0.086*
Knowledge of ART	163	81 (50)	82 (50)	0.347	167	58 (35)	109 (65)	0.357
Knowledge of ART side effects	157	80 (51)	77 (49)	0.483	161	53 (33)	108 (67)	0.044**
No fear of ART	151	73 (48)	78 (52)	0.111	154	53 (34)	101 (65)	0.336
ART adherent	166	84 (51)	82 (49)	0.496	170	61 (36)	109 (64)	0.269

Data source: field data (2021)

^***^p < 0.01, **p < 0.05, *p < 0.1 (1-sided Fisher's exact test)

HIV status disclosure to a partner was more likely to occur among married persons (Coef. = 1.25, p < 0.001, [95% CI 0.74, 1.76]), but not with non-partners (Coef. = −0.56, p < 0.001, [95% CI −0.044, 0−0.076]) (see Table 3).

**Table 3:** Bivariate probit regression on predictors of HIV status disclosure

Independent variables	Disclosure to partner (Module 1)			Disclosure to others (Module 2)		
	Coef	[95%	CI]	Coef	[95%	CI]
Sex						
Female	−0.511	−1.161	0.138	0.160	−0.441	0.762
Male	Ref	Ref	Ref	Ref	Ref	Ref
Marital status						
In a relationship with partner	1.246***	0.737	1.755	−0.560**	−1.044	−0.076
Otherwise	Ref	Ref	Ref	Ref	Ref	Ref
Religion						
Pentecostal	0.086	−0.432	0.604	−0.040	−0.535	0.455
Other religions	Ref	Ref	Ref	Ref	Ref	Ref
Education						
Primary	−0.102	−0.602	0.399	−0.020	−0.497	0.458
Higher education	Ref	Ref	Ref	Ref	Ref	Ref
Location						
Urban	0.038	−0.452	0.529	−0.049	−0.518	0.420
Rural	Ref	Ref	Ref	Ref	Ref	Ref
Constant	−**0.242**	−**0.953**	**0.469**	**0.519**	−**0.169**	**-0.161**
athrho	−0.497***	−**0.834**	−**0.161**			
Mean dependent var	0.633					
SD dependent var	0.484					
Number of obs	120					
Chi-square	30.105					
Akaike crit. (AIC)	304.938					
Prob > chi2	0.001					

Independent variables	Disclosure to partner (Module 3)			Disclosure to others (Module 4)		
	Coef	[95%	CI]	Coef	[95%	CI]
Income						
< GHC 500	−0.447*	−0.928	0.035	0.507**	0.011	1.004
≤ GHC 500	Ref			Ref		
Financial status						
Poor	0.099	−0.39	0.588	−0.391	−0.909	0.128
Not poor	Ref			Ref		
Occupation						
Private business	0.426**	0.012	0.841	−0.148	−0.574	0.279
Otherwise	Ref					
Employment status						
Employed	−0.084	−0.517	0.348	0.431*	-0.014	0.876
Not employed	Ref			Ref		
Constant	**0.129**	−**0.31**	**0.568**	**0.212**	**-0.232**	**0.656**
athrho	−0.289**	−0.563	−0.015			
Mean dependent var	0.654					
SD dependent var	0.477					
Number of obs	153					
Chi-square	0.029					
Akaike crit. (AIC)	407.852					
Prob > chi2	0.029					

Independent variables	Disclosure to partner (Module 5)			Disclosure to others (Module 6)		
	Coef	[95%	CI]	Coef	[95%	CI]
Divorced due to HIV						
Yes	−1.339***	−1.773	−0.904	0.485**	0.06	0.911
No	Ref	Ref	Ref	Ref	Ref	Ref
Place of ART attendance						
ART clinic	0.07	−0.446	0.586	0.351	−0.136	0.838
Otherwise	Ref	Ref	Ref	Ref	Ref	Ref
Knows ART side effects						
Yes	−0.31	−1.341	0.722	0.519	−0.339	1.377
No	Ref	Ref	Ref	Ref	Ref	Ref
Constant	0.942*	−**0.134**	**2.019**	−**0.547**	−**1.459**	**0.364**
athrho	−0.25*					
Mean dependent var	0.664					
SD dependent var	0.474					
Number of obs	152					
Chi-square	44.779					
Akaike crit. (AIC)	370.920					
Prob > chi2	0.000					

Data source: field data (2021)

***p < 0.01, **p < 0.05, *p < 0.1

Persons in lower income brackets were less likely to disclose their HIV status to their partners than those who earn higher monthly incomes (Coef. = −0.45, p < 0.01, [95% CI −0.93, 0.035]) (see Table 3). Higher monthly income earners were more likely to disclose their HIV status to others (non-partners) than low-income earners (Coef. = 0.51, p < 0.005, [95% CI 0.011, 1.00]) (see Table 3).

Persons engaged in private businesses were more likely to disclose their HIV status to their partners than other forms of employment endeavours (Coef. = 0.43, p < 0.005, [95% CI 0.012, 0.84). Persons who were employed were more likely to disclose their HIV status to others (non-partners) than the unemployed (Coef. = 0.43, p < 0.01, [95% CI −0.014, 0.88]) (see Table 3). Finally, persons who were divorced due to HIV were less likely to voluntarily disclose their HIV status to partners (Coef. = −1.34, p < 0.001, [95% CI −1.77, −0.90]) but more likely to disclose their status to others (non-partners) (Coef. = 0.49, p < 0.005, [95% CI 0.06, 0.91]) (see Table 3).

## Discussion

Ghana, like many HIV endemic countries in Africa, aims to eliminate new HIV infections especially among children by 2020 [22]. It was found in this study that 42% of the PLHIV indicated their partner did not test positive for HIV. This finding could be due to effective practice of safe sex or perhaps respondents did not truly know their partners status, yet gave a socially desirable response. Moreover, the finding that 91% of the PLHIV were on the ART corroborates the Ghana AIDS Commission (GAC) [5] statistics that 99% of PLHIV are on sustained ART.

An equal proportion (50%) of respondents said they have voluntarily disclosed their HIV status, contrary to 79% disclosure rate among PLHIV in an earlier study conducted in Ghana by Adam et al. [1]. However, it must be clarified that Adam et al. [1] did not distill the responses into categories of family members as examined in this paper.

It was observed that disclosures were predominantly made to non-family members (65%), corroborating earlier studies in Kenya [13], Zimbabwe [8], South Africa [12], Uganda Kairania et al. [7] and other African countries [11]. Perhaps due to stigma, most respondents felt more secured with non-family members for the needed psycho-social support, as confirmed in a study by Mokgatle et al. [12] in South Africa where almost half (45.7%) of the 670 respondents were unwilling to care for family members diagnosed of HIV/AIDS. Mistrust for family members by PLHIV could account for this perception.

Predictors of HIV status disclosure was also explored and it was found that disclosure tendencies were significantly correlated with marital status, educational level, divorce status, monthly income, occupation and having an HIV positive partner. These findings are supported by similar studies in Ghana [1], other African countries [21] and in Canada [9] where socio-economic factors were found be important correlates of HIV status disclosure. These empirical findings further buttress arguments in the literature that HIV epidemiology and disease coping mechanisms have strong socio-economic and gender underpinnings [21].

Similarly, previous studies have alluded to the strong correlation between economic freedom/self-dependence and health seeking behaviour [10]. HIV/AIDS disproportionally affects more women than men in many African countries including Ghana. As demonstrated in this study, respondents in the high-income bracket (mostly men) were more likely to disclose their HIV status to their partner than their female counterparts as found in Poku et al. [18]. Financial insecurity and fear of divorce with its unpleasant consequences perhaps explain why persons in low-income bracket are reluctant to be the first to disclose their HIV status. Finally, respondents who were found to be adherent to ART also had higher odds of disclosing their HIV status to someone. Studies have showed that persons who are adherent to ART also turn have positive outlook of HIV/AIDS and are more likely to disclose their status to others Nichols et al. [14]) [19]. This observation corroborates findings in previous studies that found that non-adherents of ART are sometimes in perpetual denial stage and not willing to seek treatment and social support in light of their condition Nichols et al. [15] [4]. It is important efforts are intensified to ensure enhanced support systems (including stigma control) for PLHIV. In conclusion, findings of this study could guide policy actors in designing HIV status disclosure support systems for PLHIV in low-and-middle-income countries (LMICs) with already fragile health systems not resilience enough to support PLHIV in this disclosure conundrum.

## Limitations

Responses were self-reported without independent verification for truth. Reponses are therefore subject to biases including socially desirable responses given the sensitive nature of the topic. However, the deployment of robust sampling techniques and reliability tests guarantee the results trustworthiness.

## Conclusion/policy recommendations

There is the need for policy debates to inform guidelines for HIV status self-disclosure support for PLHIV. Even though guidelines exist on disclosure for minors, there is no tailored framework for self-disclosure among the adult population who record higher prevalence rates. Moroever, since HIV status disclosure has gender and socio-economic underpinnings, there should be accelerated pre- and post-disclosure support system for PLHIV to ameliorate their plight.

## Data Availability

All data generated or analyzed during this study are included in this published article and its supplementary information files.

## Abbreviations

AIDS: Acquired immune-deficiency syndrome
ART: Antiretroviral therapy
COVID-19: Coronavirus disease 2009
GAC: Ghana AIDS Commission
GHS: Ghana Health Service
HIV: Human immune virus
ISAT: Internalized Stigma of AIDS Tool
LMICs: Low- and middle-income countries
MoH: Ministry of Health
NACPL: National AIDS control programme
PLHIV: People living with HIV
PMTCT: Prevention of Mother to Child Transmission
REC: Research Ethics Committee
SDGs: Sustainable development goals
TPB: Theory of planned behavior
UCSF: University of California San Francisco
UHAS: University of Health and Allied Sciences
UN: United Nations
UNAIDS: United Nations AIDS
VIFs: Variance inflation factors
ZDHS: Zimbabwe Demographic Health Survey
